# Roles of Non-Coding RNAs in Cervical Cancer Metastasis

**DOI:** 10.3389/fonc.2021.646192

**Published:** 2021-03-11

**Authors:** Tanchun Cheng, Shouguo Huang

**Affiliations:** Department of Obstetrics and Gynecology, Affiliated Haikou Hospital, Xiangya Medical College of Central South University, Haikou, China

**Keywords:** non-coding RNAs, long non-coding RNAs (lncRNAs), circular RNAs (circRNAs), cervical cancer, metastasis

## Abstract

Metastasis remains to be a huge challenge in cancer therapy. The mechanism underlying cervical cancer metastasis is not well understood and needs to be elucidated. Recent studies have highlighted the diverse roles of non-coding RNAs in cancer progression and metastasis. Increasing numbers of miRNAs, lncRNAs and circRNAs are found to be dysregulated in cervical cancer, associated with metastasis. They have been shown to regulate metastasis through regulating metastasis-related genes, epithelial-mesenchymal transition, signaling pathways and interactions with tumor microenvironment. Moreover, miRNAs can interact with lncRNAs and circRNAs respectively during this complex process. Herein, we review literatures up to date involving non-coding RNAs in cervical cancer metastasis, mainly focus on the underlying mechanisms and highlight the interaction network between miRNAs and lncRNAs, as well as circRNAs. Finally, we discuss the therapeutic prospects.

## Introduction

Cervical cancer is a common gynecological cancer which ranks fourth for both incidence and mortality in women worldwide (1). Over the last few decades, there has been a decline in incidence and mortality rates in many populations worldwide, owing to the screening techniques and application of HPV vaccines (2). However, in some developing countries, cervical cancer ranks second or remains the most frequently diagnosed cancer and the leading cause of cancer death among women (3). Furthermore, the prognosis is very poor for patients with advanced or metastatic cervical cancer, whereas current treatment is rather limited (4–6). Therefore, there is an urgent need to decipher the mechanism of metastasis and find new therapeutic targets. Clinically, cervical cancer cells usually invade adjacent tissues and metastasis through lymphatic or blood vessels to distant sites. Over the last decades, studies on mechanisms underlying cervical cancer metastasis focused mainly on oncogenes and protein-related signaling pathways (7, 8). However, the leading regulator of this process is still not well understood. Metastasis is a complex process including several steps and large amounts of molecular interactions (9). Cancer cells need to undergo phenotype changes during this complicated process. In recent years, our understanding of the non-coding transcripts has been largely advanced through high-throughput sequencing technology. Increasing evidence is pointing toward non-coding RNAs as important regulators in many aspects of cancer metastasis (10, 11).

Non-coding RNAs (NcRNAs) are transcripts that do not code for proteins, which can be roughly divided into small non-coding RNAs (smaller than 200 nt) and long non-coding RNAs (lncRNAs, longer than 200 nt) (12, 13). NcRNAs account for the majority of transcriptome, interestingly the amount of ncRNAs correlates with organismal complexity (14). Emerging evidence showed that ncRNAs have regulatory roles in diverse cellular processes both in biological and pathological conditions including cancer (15). Their roles in cancer progression and metastasis are being appreciated (16, 17). Among them, microRNAs (miRNAs), lncRNAs and circular RNAs (circRNAs) are actively studied in recent years. Research is accelerating to decipher the underlying mechanism of ncRNA-regulated cervical cancer metastasis (18, 19). In this review, we summarize the recently identified ncRNAs in cervical cancer metastasis, describe the mechanism of action and discuss their therapeutic perspectives. Particularly, we show the interaction networks between miRNAs and lncRNAs, as well as circRNAs. We hope to provide insights into the aspect of ncRNAs-regulated metastasis and their potential as therapeutic targets.

## MicroRNAs in Cervical Cancer Metastasis

MiRNAs are a class of conserved small endogenous RNAs defined as single-stranded RNAs of ~22 nucleotides in length with no protein-coding potential. Thousands of miRNAs have been identified and annotated among different species (20). Since the discovery of miRNAs over two decades ago, the biology of miRNAs has been extensively reviewed. Most miRNA genes are transcribed by RNA polymerase II (Pol II) and processed in the nucleus, then cleaved in the cytoplasm and incorporated into Argonaute protein, formulating the RNA-induced silencing complex (miRISC) containing the mature miRNA strand (21). MiRNAs are critical regulators of gene expression, they can guide miRISCs to target mRNAs by base pairing with the 3′ untranslated regions (UTRs) of mRNAs, resulting in degradation or translational repression of the mRNA targets (22). Both the 5’ and 3’ regions of miRNA provide information for the specific target recognition (23). Computational approaches to predict miRNA targets revealed that a single miRNA can target several mRNAs and a single mRNA can be regulated by different miRNAs (20). Since the early finding in 2004 revealed that nearly one-half of miRNA genes are located in fragile sites or in caner-associated genomic regions (24), a great number of miRNAs have been reported to be dysregulated in cancer with pro- or anti-tumor potential (25). Over the past decade, a series of miRNAs have been found to be aberrantly expressed in cervical cancer and correlate with metastasis (**Table 1**).

**Table 1:** Roles of miRNAs in cervical cancer metastasis.

MiRNAs	Function in metastasis	Mechanism of action	Reference
miR-21	Promote	Targe RASA1	(26, 27)
miR-221-3p	Promote	Target THBS2	(28)
miR-199b-5p	Promote	Target KLK10	(29)
miR-29a	Inhibit	Modulate methylation of SOCS1	(30)
miR-543	Inhibit	Target TRPM7	(31)
miR-106b	Promote	Target DAB2	(32)
miR-519d	Promote	Target Smad7	(33)
miR-218-5p	Inhibit	Target LYN/NF-*κ*B signaling pathway	(34)
miR-200b	Inhibit	Inhibit EMT	(35)
miR-484	Inhibit	Target ZEB1/SMAD2	(36)
miR-145	Inhibit	Inhibit EMT *via* targeting SIP1	(37)
miR-211	Inhibit	Inhibit EMT *via* targeting MUC4	(38)
miR-183	Inhibit	Target MMP9	(39)
miR-124	Inhibit	Inhibit angiogenesis *via* targeting AmotL1	(40)
miR-221-3p	Promote	Promote angiogenesis *via* targeting THBS2/MAPK10	(41, 42)

### MicroRNAs Regulate Metastasis-Related Genes

Detection of circulating miRNAs in serum of cervical cancer patients found that miR-21 was related to lymph node metastasis by inhibiting RASA1 (26). Subsequent *in vivo* study also found that miR-21 could promote lymph node metastasis in orthotopic xenograft mouse model of cervical cancer (27). Thrombospondin-2 (THBS2) is a matricellular protein with antiangiogenic activity, which can modulate extracellular matrix assembly (43), and correlates with cancer metastasis (44). In cervical cancer, miR-221-3p was found to be upregulated by the transcription factor twist2, and promote lymph node metastasis *via* targeting THBS2 (28). Another miRNA 199b-5p was reported to promote metastasis in cervical cancer by downregulating kallikrein-related peptidase 10 (KLK10) (29). Metastasis-inhibiting miRNAs have also been documented, such as miR-29a inhibits invasion and metastasis of cervical cancer through modulating methylation of suppressor of cytokine signaling protein 1 (SOCS1) (30), and miR-543 inhibits cervical cancer metastasis by targeting transient receptor potential melastatin 7 (TRPM7) (31).

### MicroRNAs Regulate Metastasis-Related Signaling Pathways

TGF-β signaling pathway has been reported to correlate with lymph node metastasis in cervical cancer (45). Recently, miR-106b was found to be involved in TGF-β-induced cell migration by targeting disabled homolog 2 (DAB2) in cervical carcinoma (32). Smad 7 is a negative regulator in TGF-β signaling pathway. Study showed that miR-519d facilitates progression and metastasis of cervical cancer through targeting smad7 (33). A recent study based on bioinformatic analysis found that miR-218-5p could inhibit cervical cancer cell metastasis *via* targeting LYN/NF-κB signaling pathway (34).

### MicroRNAs Regulate Epithelial-Mesenchymal Transition

Epithelial-mesenchymal transition (EMT) is a plastic and dynamic biological process orchestrating cell morphological changes, the reverse program of this process is called mesenchymal-epithelial transition (MET) (46). The roles of EMT plasticity in cancer metastasis have been actively studied and extensively reviewed (47, 48). EMT may contribute to the early stage of metastasis by conferring upon epithelium-derived cancer cells the capacity of migration and invasion, while MET is thought to be important for colonization of the disseminated cancer cells at distant sites (48). MiRNAs are emerging as crucial regulators of EMT by targeting multiple components of this program. The miR-200 family (miR-200a, -200b, -200c, -141 and -429) has been recognized as tumor suppressor miRNAs by inhibiting EMT (49, 50). MiR-200b has been reported to suppress cervical cancer cell invasion and metastasis through inhibiting EMT (35). Some other miRNAs have also been reported to modulate EMT and metastasis in cervical cancer. MiR-484 (36), miR-145 (37) and miR-211 (38) have recently been reported to inhibit EMT and invasion of cervical cancer cells *via* targeting ZEB1/SMAD2, SMAD-interacting protein 1 (SIP1) and mucin 4 (MUC4), respectively.

### MicroRNAs Regulate Tumor Microenvironment

The tumor microenvironment includes numerous types of stroma cells and the extracellular matrix. The reciprocal interactions between tumor cells and tumor microenvironment during tumor initiation and progression have long been recognized (51). Moreover, miRNAs have been revealed to regulate tumor microenvironment through different aspects (52). Matrix metalloproteinases (MMPs) are prominent extracellular proteinases which can influence the primary tumor invasion and metastasis (53). Research in cervical cancer found that miR-183 can target MMP-9 and then inhibit cellular invasion and metastasis (39). The induction of angiogenesis is indispensable for growth and metastasis of solid tumors in the tumor microenvironment (54). Research has reported that miR-124 could target angiomotin-like protein AmotL1 and then represses vasculogenic mimicry and cell motility in cervical carcinoma cells (40). Recent data shows that cancer-derived exosomes can transport miRNAs to regulate angiogenesis and invasion in cervical cancer. Cervical squamous cell carcinoma-secreted exosomal miR-221-3p has been shown to promote angiogenesis by targeting THBS2 (41). Additionally, another report implicated that the exosomal miR-221-3p promotes invasion, migration and angiogenesis in cervical cancer by decreasing MAPK10 (42).

## Long Non-Coding RNAs in Cervical Cancer Metastasis

LncRNAs are defined as transcripts longer than 200nt with no significant open reading frames and encode no proteins (55). The biogenesis of lncRNAs is much like mRNAs. Many lncRNAs are transcribed by RNA polymerase II, polyadenylated, spliced and 5′-capped, but tend to be shorter than mRNAs (56). Moreover, lncRNAs are expressed at relatively low levels compared with mRNAs, but show more cell-type and tissue-type specificity (56). LncRNAs can be roughly divided into five classes according to their location in the genome where they are transcribed, including long intergenic noncoding RNAs (lincRNAs), natural antisense transcripts, pseudogenes, long intronic ncRNAs and the ncRNAs produced from the transcription start sites, such as promoter-associated RNAs and enhancer RNAs (57).

**Figure 1:**
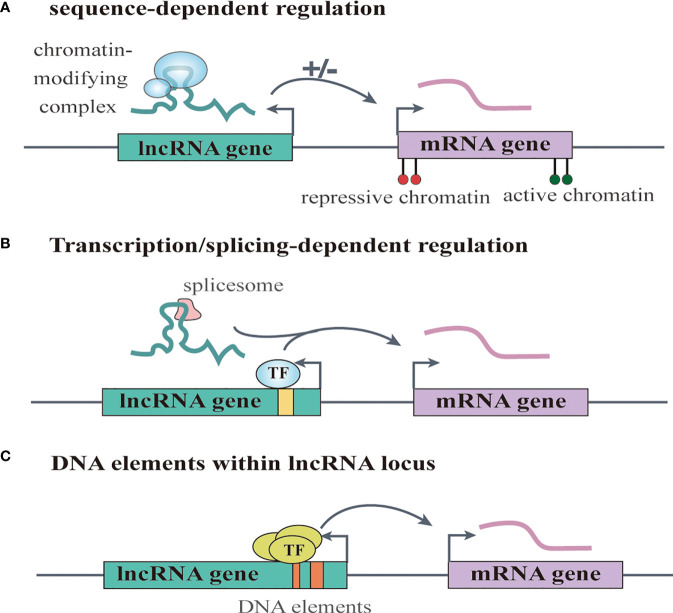
LncRNAs regulate gene expression in cis. **(A)** sequence-dependent regulation, the lncRNA transcript can recruit regulatory factors to specific gene loci. **(B)** transcription or splicing-dependent regulation, the act of lncRNA transcription rather than the transcript itself can affect gene expression. **(C)** the cis-acting DNA elements within lncRNA loci can also regulate adjacent gene expression.

**Figure 2:**
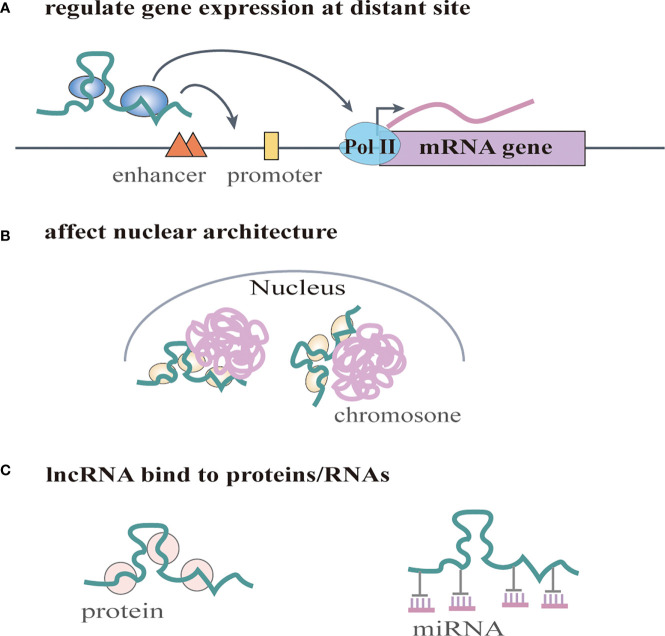
LncRNAs regulate gene expression in trans. **(A)** lncRNAs regulate gene expression at distant site by interacting with promoters, enhancers or proteins binding with these sites. They can also regulate RNA polymerase activities. **(B)** lncRNAs affect nuclear architecture to influence gene expression. **(C)** lncRNAs can bind to and regulate the activity or abundance of proteins or RNAs by function as decoys or competing endogenous RNAs (ceRNAs).

Increasing numbers of lncRNAs have been identified, but only a small fraction of them have been functionally characterized (58). Unlike miRNAs which function predominantly in the cytoplasmic compartment, lncRNAs are found both in the nucleus and the cytoplasm (59), indicating that lncRNAs may function through diverse mechanisms. LncRNAs have been shown to regulate gene expression at different levels, and they can regulate gene expression either in cis or in trans (14, 60). Cis-acting lncRNAs can regulate the expression or chromatin state of nearby genes through three common mechanisms: (1) sequence-dependent lncRNA regulation, the lncRNA transcript can recruit regulatory factors to specific gene loci (**Figure 1A**) **(**61); (2) transcription or splicing-dependent regulation, the act of lncRNA transcription rather than the transcript itself can affect gene expression (**Figure 1B**) **(**62); and (3) the cis-acting DNA elements within lncRNA loci can also regulate adjacent gene expression (**Figure 1C**) **(**63). While, the trans-acting lncRNAs can leave the site of transcription and regulate gene expression at independent sites (60). For example, they can regulate gene expression at distant sites by interacting with promoters, enhancers or proteins binding with these sites (**Figure 2A**) **(**64), or modulating chromatin states (65) and RNA polymerase activities (**Figure 2B**) **(**66). Moreover, some lncRNAs may affect nuclear architecture to influence gene expression (**Figure 2B**) **(**67). Additionally, some trans-acting lncRNAs can bind to and regulate the activity or abundance of proteins or RNAs by function as decoys or competing endogenous RNAs (ceRNAs) **(****Figure 2C**) **(**68, 69). In consideration of the diverse functions of lncRNAs, recent studies have highlighted the significant roles of lncRNAs in cancer progression (70, 71). A series of lncRNAs have been revealed to play crucial roles in cervical cancer metastasis (**Table 2**).

**Table 2:** Roles of lncRNAs in cervical cancer metastasis.

LncRNAs	Function in metastasis	Mechanism of action	Reference
MALAT1	Promote	Induce EMT	(72)
	Promote	Sponge miR-429	(73)
	Promote	Sponge miR-202-3p and upregulate periostin	(74)
EBIC	Promote	Bind to EZH2 and repress E-cadherin	(75)
TUG1	Promote	Promote EMT	(76)
CTS	Promote	Promotie EMT *via* miR-505/ZEB2 axis	(77)
HOTAIR	Promote	Sponge miR-23b and upregulate MAPK1	(78)
	Promote	Target Notch signaling pathway	(79)
	Promote	Synergiz with STAT3	(80)
Xist	Promote	Sponge miR-889-3p and upregulate SIX1	(81)
799	Promote	Sponge miR-454-3p and upregulate TBL1XR1	(82)
XLOC_006390	Promote	Sponge miR-331-3p and miR-338-3p	(83)
TTN-AS1	Promote	Sponge miR-573 and regulate E2F3	(84)
ZNF667-AS1	Inhibit	Sponge miR-93-3p and upregulate PEG3	(85)
DANCR	Promote	Sponge miR-665	(86)
PVT1	Promote	Epigenetically silence miR-200b/miR-195	(87, 88)
GAS5-AS1	Inhibit	Increase GAS5 stability by epigenetic modulation	(89)
DGCR5	Inhibit	Target WNT signaling pathway	(90)
ANRIL	Promote	Target PI3K/Akt pathway	(91, 92)

### Long Non-Coding RNAs Regulate Epithelial-Mesenchymal Transition

Some lncRNAs have been found to regulate EMT-related genes and EMT-transcription factors. Metastasis-associated lung adenocarcinoma transcript 1 (MALAT1) is a highly conserved and abundant lncRNA, which was originally described to play crucial roles in lung cancer metastasis (93). It has also been reported to associate with metastasis of many other tumors (94). In cervical cancer, study showed that MALAT1 promotes invasion and metastasis of cervical cancer cells *via* inducing EMT (72). Another lncRNA EBIC which can bind to enhancer of zeste homolog 2 (EZH2) in cervical cancer has been reported to promote cell invasion by repressing E-cadherin (75). Taurine-upregulated gene 1 (TUG1) is an oncogenic lncRNA in multiple human cancers (95). It has been found to regulate cervical cancer cells migration and invasion by promoting EMT (76). A novel lncRNA CTS identified from the lncRNA microarray database was found to promote metastasis and EMT of cervical cancer by regulating miR-505/ZEB2 axis (77).

### Long Non-Coding RNAs Regulate Metastasis by Interacting With MicroRNAs

The vast majority of transcripts in the genome can interact with each other through different mechanisms (96, 97). For example, lncRNAs can act as competing endogenous RNAs (ceRNAs) to sequester miRNAs, regulating the abundance and activity of miRNAs, leading to derepression of genes targeted by corresponding miRNAs (98, 99). The ceRNA network is also implicated in cancer progression (100).

As mentioned previously, MALAT1 is involved in cervical cancer metastasis. Recent study showed that MALAT1 can sponge miR-429 to promote cervical cancer metastasis and progression both *in vitro* and *in vivo (*73). Another study also found that MALAT1 can promote invasion of cervical cancer cells through sponging miR-202-3p and upregulating expression of periostin (74). HOX transcript antisense intergenic RNA (HOTAIR) is a trans-acting lncRNA which was originally found to promote metastasis in breast cancer by reprograming chromatin state (101). In cervical cancer, HOTAIR was reported to enhance metastatic potential by sponging miR-23b and modulating the expression of MAPK1 (78). Xist is a well-known lncRNA derived from XIST gene which can regulate X-chromosome inactivation (102), and recognized as a tumor promoter in various malignant tumors (103). It has been revealed to promote cervical cancer cell invasion and migration *via* competitively binding miR-889-3p and upregulating SIX1 (81). LncRNA 799 is a lncRNA identified from microarray analysis, which has been shown to promote metastasis of SiHa cells *via* competing for miR-454-3p and upregulating transducing β-like protein1-related protein (TBL1XR1) (82). Another microarray-identified lncRNA XLOC_006390 was found to facilitate metastasis as a ceRNA against miR-331-3p and miR-338-3p in cervical cancer (83). A novel lncRNA TTN-AS1 was found to promote metastasis of cervical cancer cells *via* sponging miR-573 and regulating E2F3 (84). LncRNA Zinc finger protein 667-antisense RNA 1 (ZNF667-AS1) was revealed to suppress metastasis in cervical cancer by sponging miR-93-3p and upregulating PEG3 (85). Another cancer-related lncRNA DANCR was found to act as a ceRNA for miR-665 and promote metastasis of cervical cancer through the ERK/SMAD pathway (86).

Besides, lncRNAs can regulate expression of miRNAs through epigenetic regulation. PVT1 is an oncogenic lncRNA involved in a variety of cancer types, correlates with the copy number of the MYC oncogene (104). Increased PVT1 expression in cervical cancer contributes to cancer phenotype and associates with poor prognosis (105). Further studies showed that PVT1 could contribute to cervical cancer progression and metastasis through epigenetically silencing miR-200b (87) and miR-195 (88) respectively and modulating EMT. Growth arrest-specific transcript 5 (GAS5) is down-regulated in several cancers and recognized as a tumor suppressing lncRNA. The antisense transcript of GAS5 (GAS5-AS1) has been reported to suppress metastasis of cervical cancer by modulating GAS5 epigenetically and increasing its stability (89).

### Long Non-Coding RNAs Regulate Metastasis-Related Signaling Pathways

LncRNAs have also been revealed to drive different cancer phenotypes through regulating the intracellular signaling networks (106). HOTAIR has been revealed to target the Notch signaling pathway (79) or synergize with STAT3 (80) to promote metastasis of cervical cancer cells. DGCR5 (Digeorge syndrome critical gene 5, also known as linc00037) is a lncRNA downregulated in Huntington’s disease neurodegeneration, which has also been implicated in cancer progression. It has been demonstrated that DGCR5 suppressed migration and invasion of cervical cancer cells by targeting WNT signaling (90). Additionally, ANRIL knockdown inhibits cell proliferation and metastasis *in vitro*, and its inhibition guides inactivation of the PI3K/Akt pathway in cervical cancer (91, 92).

### Other Long Non-Coding RNAs Involved in Cervical Cancer Metastasis

Recent findings add to a growing list of lncNRAs associated with cervical cancer metastasis, pending further mechanistic investigation. For example, Colon cancer associated lncRNA (CCAT1) was found to be highly expressed in cervical cancer, and silencing of CCAT1 led to suppression of metastasis of Hela cells (107). LncRNA ATB is a lncRNA activated by TGF-β, originally identified in hepatocellular carcinoma with critical roles in invasion-metastasis cascade (108). Later, a series of studies revealed that ATB could promote metastasis in other cancers (109, 110). Study in cervical cancer showed that ATB is upregulated in cervical cancer tissues and cell lines, and correlates with lymph node metastasis and poor prognosis (111). LncRNA small nucleolar RNA host gene 1 (SNHG1) is enriched in nuclear and found to regulate gene transcription either in cis or in trans (112). Recently, SNHG1 was reported to be highly expressed in cervical cancer and knock-down of SNHG1 decreased migration and invasion of cancer cells (113). Another nuclear-enriched transcript lncRNA NEAT1 is an essential component of paraspeckle, correlates with p53 activation and chemosensitivity (114). Study in cervical cancer showed that high expression of NEAT1 predicted poor prognosis and promoted migration and invasion of cervical cancer cells (115). Upregulation of lncRNA CCHE1 in cervical cancer is correlated with advanced FIGO stage, larger tumor size, lymph node metastasis, invasion of the uterine corpus and poor prognosis (116). Further study is needed to identify the underlying mechanism of the lncRNAs discussed above.

## Circular RNAs in Cervical Cancer Metastasis

Circular RNAs (circRNAs) are newly identified class of ncRNAs, different from linear RNAs, they are covalently closed and lack polyadenylation (117). CircRNAs can arise from exons or introns within precursor mRNAs (pre-mRNAs), formed by backsplicing or intronic lariats (118, 119). Interestingly, genome-wide analyses have shown that many circRNAs are abundant, highly stable and evolutionarily conserved in mammalian cells (120, 121). However, biological functions of circRNAs still need further investigation. It has been suggested that circRNAs can function as miRNA or protein sponges, regulate transcription of parental genes in cis, even under some circumstances circRNAs can be translated (119, 122). In recent years, roles of circRNAs in cancer are emerging (123). In cervical cancer, several circRNAs have been implicated in cancer metastasis (**Table 3**).

**Table 3:** Roles of circRNAs in cervical cancer metastasis.

LncRNAs	Function in metastasis	Mechanism of action	Reference
Circ-0000745	Promote	Regulate E-cadherin	(121)
Circ-000284	Promote	Sponge miR-506 and regulate Snail-2	(122)
circ-NRIP1	Promote	Sponge miR-629-3p and targe PTP4A1/ERK1/2 pathway	(123)
Circ-0003204	Promote	Regulate MAPK pathway	(124)
circUBAP2	Promote	Modulate miR-361-3p/SOX4 axis	(125)

High-throughput sequencing technology has been employed to explore circRNA expression profile in cervical cancer tissues and cell lines. A couple of circRNAs have been reported to regulate metastasis through EMT and interaction with signaling pathways. Circ-0000745 was found to be upregulated in cervical cancer tissues, associated with poor differentiation and vascular/lymphatic invasion. Inhibition of circ-0000745 led to down regulation of E-cadherin and reduced migration and invasion (124). Circ-000284 was found to be upregulated in cervical cancer cells. Loss of function assay showed that circ-000284 can promote proliferation and invasion of cervical cancer *via* sponging miR-506 and regulating Snail-2 (125). Another upregulated circRNA circ-NRIP1 was reported to be relevant to lymphovascular space invasion. Mechanistic investigation showed that circ-NRIP1 can promote migration and invasion of cervical cancer by sponging miR-629-3p and targeting the PTP4A1/ERK1/2 pathway (126). Circ-0003204 was identified by RNA sequencing, which was also upregulated in cervical cancer, found to promote proliferation, migration and invasion of cervical cancer cells through regulating MAPK pathway (127). Recently, the roles of circUBAP2 have been implicated in different cancers. In cervical cancer, it was found to be upregulated and contribute to tumor growth and metastasis by modulating miR-361-3p/SOX4 axis (128). Roles of circRNAs in cervical cancer metastasis remain largely unknown and research is increasing.

## Conclusions and Perspectives

The improvement in sequencing technology led to exploding increase in different types of ncRNAs. NcRNAs are emerging as active regulators of cellular process in cancer. MiRNAs are well studied over the past decade, lncRNAs are actively studied for their diverse roles in gene expression regulation, circRNAs are recently identified and their functions need further investigation. Besides, the ncRNAs themselves can interact with each other, such as lncRNAs and circRNAs can sponge miRNAs. The RNA molecules sharing miRNA response elements (MREs) can act as ceRNAs and crosstalk with each other, indicating an enormous and complex regulatory network orchestrating cellular process. As described above, a lot of ncRNAs are dysregulated in cervical cancer, exhibiting either metastasis-promoting or -inhibiting roles through different ways. These findings give insights into target therapy based on RNAs.

Given the diverse roles of ncRNAs in cervical cancer metastasis and their highly expression specificity, therapeutics targeting these regulatory ncRNAs may be appreciated. Overexpression and knock-down approaches used in experimental studies to modulate gene expression and metastasis have shown promising results. Indeed, therapeutic approaches targeting RNAs such as small interfering RNA (siRNA) and antisense oligonucleotide (ASO) have been exploited for many years, as reviewed elsewhere (129–131). Progress is gratifying for more than 100 RNA-targeted therapies have reached clinical development and some have been approved for commercial use in rare disease or chronic disease (132, 133). At present, some phase 1 clinical trials of ASO-based drugs in advanced metastatic cancer treatment are undergoing (e.g., NCT00466583, NCT01120288 and NCT00471432).

The field of ncRNAs in cancer metastasis is promising, which is progressing rapidly for the new classes of ncRNAs are emerging. Large amount of experimental work is still needed to decipher the mechanisms underlying metastasis and fully assess their therapeutic potential. With the development of nucleic acid therapeutics, we hope to identify ncRNAs which can be targeted in advanced and metastatic cervical cancer patients.

## Author Contributions

TC performed the literature search and wrote the manuscript. SH reviewed and edited the manuscript. All authors contributed to the article and approved the submitted version.

## Conflict of Interest

The authors declare that the research was conducted in the absence of any commercial or financial relationships that could be construed as a potential conflict of interest.
